# Risk Assessment and Epidemiologic Evidence in Environmental Health Science

**DOI:** 10.1289/ehp.114-a634b

**Published:** 2006-11

**Authors:** Bernard D. Goldstein

**Affiliations:** Graduate School of Public Health, University of Pittsburgh, Pittsburgh, Pennsylvania, E-mail: bdgold@pitt.edu

There appears to be a serious conceptual error about the role of the various environmental health sciences in Kundi’s otherwise interesting and informative commentary on “Causality and the Interpretation of Epidemiologic Evidence” (Kundi 2006). This error is exemplified in his next-to-last paragraph:

Most risk assessment procedures demand that for chronic diseases such as cancer there must be epidemiologic evidence before an extrinsic agent can be ascribed a hazardous potential for human health.

In fact, it is solely toxicologic evidence that is used for the overwhelming majority of agents to which a “hazardous potential for human health” is ascribed. I am unaware of any risk assessment process that requires epidemiology to recognize hazardous potential for human health.

Perhaps Kundi (2006) meant that there must be epidemiologic evidence for a chemical to achieve the level of a known or proven cause of a hazard to human health. However, the misunderstanding in the above quote permeates his commentary.

As Kundi (2006) correctly recognized, it is better to prevent the introduction or use of agents that would cause adverse effects eventually identifiable in an epidemiologic study. Such prevention is primarily the role of predictive toxicology. Yet, as Kundi stated in his abstract, his recommended dialogue approach to “the potential for disease causation” starts with epidemiology.

Kundi (2006) concluded that the principle that every disease has a cause is metaphysical, but still has heuristic value. He appears to mean that the principle of causation helps us explore the potential that environmental factors cause human disease—and that we do so by developing models, such as risk assessment, that approximate reality without achieving certainty. However, a risk assessment, or any other model, that must depend on epidemiologic evidence to recognize the potential for disease causation represents a failure of environmental health science.
